# Glucocorticoids Induce an Opposite Metabolic Switch in Human Monocytes Contingent upon Their Polarization

**DOI:** 10.3390/biom15101422

**Published:** 2025-10-07

**Authors:** Elisa Peruzzi, Sophia Heidenreich, Lucas Klaus, Angela Boshnakovska, Agathe Amouret, Tobias Legler, Sybille D. Reichardt, Fred Lühder, Holger M. Reichardt

**Affiliations:** 1 Institute for Cellular and Molecular Immunology, University Medical Center Göttingen, Humboldtallee 34, 37073 Göttingen, Germany; elisa.peruzzi@med.uni-goettingen.de (E.P.); sophia.heidenreich@med.uni-goettingen.de (S.H.); lucas.klaus1@med.uni-goettingen.de (L.K.); agathe.amouret@med.uni-goettingen.de (A.A.); sybille.reichardt@med.uni-goettingen.de (S.D.R.); 2 Institute of Cellular Biochemistry, University Medical Center Göttingen, Humboldtallee 23, 37073 Göttingen, Germany; angela.boshnakovska@med.uni-goettingen.de; 3 Department of Transfusion Medicine, University Medical Center Göttingen, Robert-Koch-Strasse 40, 37075 Göttingen, Germany; tlegler@med.uni-goettingen.de; 4 Institute for Neuroimmunology and Multiple Sclerosis Research, University Medical Center Göttingen, Von-Siebold-Strasse 3a, 37075 Göttingen, Germany; fred.luehder@med.uni-goettingen.de

**Keywords:** glucocorticoids, monocytes, human, glycolysis, oxidative phosphorylation

## Abstract

**Background:** Monocytes can commit to different phenotypes associated with specific features required in inflammation and homeostasis. Classical and alternative activation are two extremes of monocyte polarization and are both influenced by glucocorticoids (GCs). **Methods:** Human monocytes were sorted from the blood of healthy individuals and activated with LPS or IL-4 and IL-13, either in the absence or presence of dexamethasone (Dex). Metabolic adjustments were analyzed using Seahorse stress tests, SCENITH, and RT-qPCR. **Results:** LPS enhanced glycolysis and also, to a lesser extent, oxidative phosphorylation (OXPHOS), whereas addition of Dex induced a metabolic switch in favor of the latter. In contrast, activation of monocytes with IL-4 and IL-13 exclusively stimulated OXPHOS, which was suppressed by concomitant Dex treatment. The glycolytic function of monocytes matched alterations in gene expression of glucose transporters and metabolic enzymes, which were upregulated by LPS and inhibited by Dex via interference with the mTORC1 pathway but remained unaltered in response to IL-4 and IL-13. Although the dependency of classically and alternatively activated monocytes on OXPHOS and glucose usage markedly differed, modulation by GCs was limited to the latter polarization state. **Conclusions:** Our findings unravel a highly selective regulation of human monocyte energy metabolism by different activating stimuli as well as by GCs.

## 1. Introduction

Glucocorticoids (GCs) are highly potent modulators of innate and adaptive immune responses and remain indispensable for the clinical management of many inflammatory diseases [1]. They regulate multiple leukocyte subsets including T cells and myeloid cells through a plethora of mechanisms including induction and repression of cytokines, chemokines, adhesion molecules as well as cytotoxic mediators. Furthermore, GCs influence proliferation, differentiation, and survival of immune cells [2]. Control of immunometabolism, however, has only recently gained interest again, although cortisol had already been shown to inhibit glucose transport in T cells half a century ago [3].

Monocytes develop in the bone marrow and migrate to sites of inflammation along chemotactic cues where they not only replenish tissue-resident macrophages but also contribute to tissue homeostasis and pathology [4]. In response to a variety of stimuli, monocytes and their descendants commit to different phenotypes that comply with the broad spectrum of tasks that these cells fulfill in physiological processes [4,5,6]. Notably, monocytes exhibit greater responsiveness to GCs than macrophages, reflecting a highly dynamic transcriptomic profile that facilitates phenotypic plasticity [7]. Considering the long duration of macrophage differentiation, one can assume that monocytes polarized in response to stimuli from the microenvironment play important autonomous roles, especially in the early phase of inflammation.

Classical activation by LPS induces a pro-inflammatory phenotype in monocytes and macrophages, which is characterized by an extensive production of TNFα, IL-1β, and IL-6 governing the acute-phase response and the secretion of reactive oxygen species as well as nitric oxide contributing to efficient pathogen clearance [5,8,9]. Additionally, enhanced expression of CD80 and IL-12 promotes an adaptive Th1 response. Alternative activation of monocytes and macrophages is induced by IL-4 and IL-13, both produced by Th2 cells. As a consequence, the mannose receptor CD206 is upregulated, thereby stimulating the scavenging of cell debris and apoptotic vesicles [10]. Expression of arginase 1 counteracts nitric oxide production and catalyzes ornithine synthesis, thereby reinforcing fibroblast proliferation and collagen deposition involved in wound healing and tissue repair [11]. In the presence of IL-10 and GCs, monocytes and macrophages adopt a deactivated phenotype, which represents an independent polarization state [5]. It is characterized by reduced levels of MHC class II molecules and pro-inflammatory cytokines and induction of CD163, which serves to remove toxic hemoglobin–haptoglobin complexes [12]. This concept also aligns with the observation that GCs promote monocyte differentiation to a macrophage phenotype characterized by high levels of CD163 and CD206 and the capacity to support erythropoiesis [13]

Pro-inflammatory monocytes and macrophages need large amounts of ATP at their disposal to meet their energy demand for the synthesis of cytokines and microbicidial molecules [14,15]. Therefore, they strongly rely on glycolysis, a rapid although inefficient pathway of energy production [16]. In contrast, polarization to an anti-inflammatory phenotype is associated with a central role of oxidative phosphorylation (OXPHOS), whereas the contribution of glycolysis is less important [17]. HIF1α is a master regulator of energy metabolism in cells that have adopted a pro-inflammatory phenotype and contributes to the enhanced glucose transport and an upregulation of glycolytic enzymes such as HK2 and PFKFB3 [18,19]. Reinforcement of glycolysis following classical activation further involves signaling via the mTORC1 pathway [20]. Under these conditions, the majority of the pyruvate generated by the breakdown of glucose is reduced to lactate and excreted instead of being further metabolized in the mitochondria. Noteworthy, metabolic processes in the context of alternative activation have not been fully resolved [21,22].

The glucocorticoid receptor (GR) is a ligand-activated transcription factor that resides in an inactivate state in the cytosol complexed with heat-shock proteins. GCs can passively cross the plasma membrane and induce a conformational change in the GR, thus resulting in its nuclear translocation [2]. Recognition of GC response elements located in a variety of genes then results either in the activation or repression of gene transcription. Alternatively, the GR can interact with signaling pathways in the cytosol, thereby indirectly impacting gene expression. Notwithstanding the great variety of molecular mechanisms of the GR, the influence of GCs on immunometabolism is only poorly understood [15,23]. We recently provided evidence that GCs control glucose transport and glycolysis in T cells [24]. Activation in the presence of Dex resulted in a diminished import of glucose, the suppression of glycolytic enzymes, and a reduced lactate production. These effects could be assigned to a blockade of mTOR phosphorylation, also highlighting the importance of non-genomic mechanisms of GCs for T cell metabolism [24]. Analysis of multiple sclerosis patients confirmed that GC therapy similarly resulted in a suppression of the glycolytic function of T cells in vivo [24]. Insights into the control of macrophage metabolism have been reported too, although the majority of the data are based on the analysis of murine cells. LPS was consistently found to enhance the glycolytic function of macrophages, an effect which was counteracted by Dex and is likely to involve HIF1α. In contrast, regulation of OXPHOS by LPS remains controversial and seems to differ between species [25,26,27,28]. Interestingly, GCs were shown to reinforce OXPHOS in murine bone marrow-derived macrophages by increasing the activity of pyruvate dehydrogenase via non-genomic mechanisms, thus uncovering mitochondrial respiration as a potential rheostat of GC activity [25]. It is noteworthy that immunometabolism of alternatively activated monocytes and macrophages and its control by GCs have not been explored.

Understanding how GCs selectively modulate the energy metabolism of classically and alternatively activated monocytes could help predict or enhance GC efficacy, design tailored therapies for inflammatory diseases, sepsis, and cancer patients, and develop combinational treatment strategies aimed at reducing GC dosage and limiting adverse effects. In this study, we thus activated human monocytes either classically (via LPS) or alternatively (via IL-4 and IL-13) and could thereby show that GCs influence their metabolism differently. In classical activation, Dex induced a metabolic switch in favor of OXPHOS by repressing glycolysis via the mTORC1 pathway. In alternative activation, Dex exclusively inhibited OXPHOS, resulting in an opposite metabolic switch. Hence, GCs influence the energy metabolism of monocytes dependent on their polarization.

## 2. Materials and Methods

### 2.1. Isolation of Human Monocytes

Density gradient centrifugation was used to isolate PBMCs from buffy coats prepared from the peripheral blood of anonymous healthy individuals as described previously [24]. Monocytes were purified using the EasySep^TM^ human monocyte enrichment Kit (Stemcell Technologies, Cologne, Germany) according to the manufacturer’s instructions. The purity of the cell preparations was confirmed by flow cytometric analysis and was routinely found to be >85%.

### 2.2. Cell Culture Experiments

Monocytes were seeded in 24-well suspension culture plates at a density of 2 × 10^6^ cells/mL in a volume of 250 µL of RPMI-1640/GlutaMAX medium supplemented with 10% FCS and 1% penicillin/streptomycin (ThermoFisher, Osterode, Germany) and incubated for 20 h at 37 °C and 5% CO_2_. Stimulation was achieved either by adding 10 ng/mL LPS (lipopolysaccharide E. coli O55:B5; Sigma-Aldrich, Darmstadt, Germany) or 50 ng/mL recombinant human IL-4 and IL-13 each (Peprotech, Osterode, Germany). Dex-phosphate was added at a final concentration of 10^−7^ to 10^−5^ M and rapamycin at a final concentration of 20 nM (both Sigma-Aldrich), each for the entire incubation period. On the next day, the slightly adherent monocytes were harvested by repeated vigorous pipetting and then separated from the cell culture supernatant by centrifugation. The survival rate of the monocytes after culturing was routinely >90% based on flow cytometric analysis.

### 2.3. Flow Cytometry

Extracellular staining was performed with fluorochrome-labeled monoclonal antibodies obtained from Biolegend (Uithoorn, The Netherlands; clone name in brackets): anti-hHLA-DR (1.243), anti-hCD14 (HCD14), anti-hCD25 (BC96), anti-hCD80 (W17149D), anti-hCD163 (GHI/61), and anti-hCD206 (15-2). Intracellular staining of phosphorylated mTOR and STAT6 as well as peptidyl puromycin was performed with the help of reagents from BD Biosciences (Heidelberg, Germany) according to our previously published protocol [29] using the following antibodies: anti-pmTOR-Ser2448 (MRRBY; ThermoFisher), anti-pSTAT6-Tyr641 (CHI24N; ThermoFisher), and anti-puromycin (2A4; Biolegend). Cells were analyzed with the help of a FACSCantoII flow cytometer (BD Biosciences) and the FlowJo^®^ software (version 10.7, Treestar, Ashland, OR, USA).

### 2.4. Seahorse Experiments

The Seahorse XF Glycolysis Stress Test Kit User Guide (103020-400, Agilent Technologies, Waldbronn, Germany) was followed to evaluate the extracellular acidification rate (ECAR) using a Seahorse XF96e Extracellular Flux Analyzer. To this end, 375.000 cells were seeded in quadruplicate in a Seahorse plate employing XF RPMI medium (pH 7.4) supplemented with 1 mM pyruvate and 2 mM glutamine, and the initial acidification of the media was assessed. Subsequent measurements to analyze glycolysis and the maximal glycolytic capacity were performed after sequential addition of 10 mM glucose and 3 μM oligomycin following a predefined time schedule. The final addition of 50 mM 2-deoxy-D-glucose (2-DG) was used as a negative control. To determine the oxygen consumption rate (OCR), the Seahorse XF Cell Mito Stress Test Kit User Guide (103016-400, Agilent Technologies) was followed. Again, 375.000 cells were seeded in a Seahorse plate in quadruplicate by using XF RPMI medium (pH 7.4) supplemented with 1 mM pyruvate, 2 mM glutamine, and 10 mM glucose. After measuring baseline respiration, 3 μM oligomycin, 1.5 μM CCCP, and 0.5 μM rotenone/antimycin A were added sequentially. The OCR, including maximal respiration, was assessed with a predefined time schedule.

### 2.5. Lactate Assay

The amount of L-lactate in the medium was determined using a commercially available kit (Cayman Chemical, Ann Arbor, MI, USA) as described previously [30]. In brief, cell culture supernatants were deproteinated with metaphosphoric acid and subsequently neutralized. The salts were precipitated and the samples placed in a black 96-well plate. L-lactate was enzymatically converted to pyruvate, yielding a fluorescent product that was detected with an excitation wavelength of 530–540 nm and an emission wavelength of 585–595 nm using an Infinite 200 Pro reader (Tecan, Männedorf, Switzerland).

### 2.6. SCENITH Assay

Metabolic changes at the single-cell level were determined by using SCENITH (single-cell energetic metabolism by profiling translation inhibition), a method which is based on the covalent binding of puromycin to nascent polypeptides. Premature termination of translation results in the release of peptidyl puromycin, which can be detected by intracellular flow cytometry and serves as a surrogate of ATP production [31]. To selectively inhibit individual metabolic pathways, 2-DG (100 mM; Sigma-Aldrich), oligomycin (1 µM; Sigma-Aldrich), or both were added to individual wells. Untreated cells served as a control. After 15 min, puromycin dihydrochloride (2 µg/mL; Santa Cruz Biotechnology, Dallas, TX) was added, and the cells were incubated for another 30 min. Extracellular staining with anti-hHLA-DR and intracellular staining with anti-puromycin were performed and analyzed by flow cytometry, as described above.

### 2.7. Quantitative RT-PCR

Total RNA was prepared with a Quick-RNA MiniPrep Kit (Zymo Research, Freiburg, Germany) and subsequently reverse-transcribed into cDNA using an iScript kit (Bio-Rad, Munich, Germany). RT-qPCR analysis was performed on an ABI 7500 Instrument (ThermoFisher) utilizing a SYBR Green Master Mix from the same company. Relative gene expression was calculated with the ΔΔCt method using *RNA18S* as a housekeeping gene. Primers were synthesized by Metabion (Planegg, Germany), and their sequences can be found in Supplementary Table S1.

### 2.8. Statistical Analysis

Either a one-way ANOVA followed by Tukey’s multiple comparison test or a paired *t*-test were used for statistical analysis using the GraphPad Prism^®^ software (v5.04; San Diego, CA, USA). Data are depicted as the mean ± SEM. Levels of significance are as follows: *: *p* < 0.05; **: *p* < 0.01; ***: *p* < 0.001; n.s.: *p* > 0.05.

## 3. Results

### 3.1. Classical and Alternative Activation of Human Monocytes

**Figure 1:**
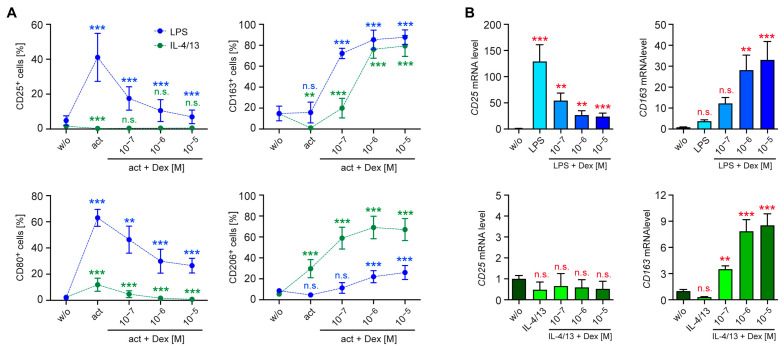
Phenotypic alterations in activated human monocytes. Monocytes were magnetically purified from buffy coats of anonymous healthy individuals and activated (act) in vitro for 20 h with LPS or IL-4 and IL-13, either in the absence or presence of 10^−7^ to 10^−5^ M Dex. Unstimulated monocytes (w/o) served as a control. (**A**) The percentage of HLA-DR^+^ CD14^+^ monocytes expressing CD25, CD80, CD163, or CD206 on the surface was determined by flow cytometry. N = 5–10. (**B**) Gene expression of *CD25* and *CD163* was analyzed by RT-qPCR using *RNA18S* as a housekeeping gene; average mRNA levels in control monocytes were set to 1. N = 14–21. All values are depicted as the mean ± SEM. Biological replicates refer to individual donors. Statistical analysis was performed by one-way ANOVA followed by Tukey’s multiple comparison test. Levels of significance (from left to right: w/s vs. act; act vs. 10^−7^ M; act vs. 10^−6^ M; act vs. 10^−5^ M): **: *p* < 0.01; ***: *p* < 0.001; n.s.: *p* > 0.05.

Monocytes can commit to distinct phenotypes which are associated with specialized functions. To establish an experimental system with which to investigate the influence of GCs on this process in vitro, we purified monocytes from the peripheral blood of healthy individuals by magnetic cell sorting and activated them with two different stimuli for 20 hrs. Classical activation was achieved by incubation with 10 ng/mL LPS, whereas the combined treatment with 50 ng/mL IL-4 and IL-13 was employed to achieve an alternative activation. In addition, monocytes were incubated with 10^−7^ to 10^−5^ M Dex added concomitantly with stimulation. The comparably high GC concentrations reflect our observation that human leukocytes are generally less sensitive to Dex than murine cells. Flow cytometric analysis revealed that surface levels of the activation marker CD25 were strongly upregulated by LPS, which was prevented by Dex in a dose-dependent manner. In contrast, treatment with IL-4/13 reduced CD25 levels even further, an effect which was not influenced by GCs (Figure 1A). The costimulatory molecule CD80 was upregulated by both stimuli, albeit to a different degree, and repressed by Dex in a dose-dependent manner (Figure 1A). CD163 surface expression was reduced in response to IL-4/13 treatment, while it was unaltered by LPS. Conversely, expression of CD206 was induced by IL-4/13 but unchanged by LPS. Importantly, surface levels of both proteins were strongly increased by Dex regardless of the type of activation (Figure 1A). Importantly, this finding aligns with a previous report on the role of GCs in monocyte differentiation [13]. Analysis of gene expression by RT-qPCR confirmed that LPS induced *CD25*, which was prevented by Dex (Figure 1B). In contrast, *CD25* was unaffected by IL-4/13. *CD163* levels were unaltered by both types of activation but strongly upregulated by Dex (Figure 1B). Taken together, our data suggest that GCs represent a genuine stimulus that conveys a highly specific monocyte phenotype.

### 3.2. Glycolytic Function of Activated Human Monocytes

**Figure 2:**
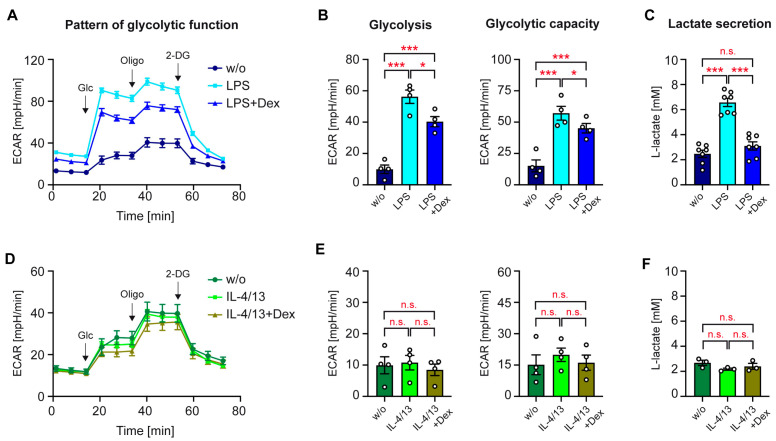
Regulation of the glycolytic function in human monocytes. Monocytes were sorted from buffy coats of anonymous healthy individuals and activated for 20 h either LPS or IL-4 and IL-13 in vitro, either in the absence or presence of 10^−5^ M Dex. Unstimulated monocytes (w/o) served as a control. (**A**,**D**) The glycolytic function was determined by measuring the ECAR using a Seahorse XF96e Analyzer after sequential addition of glucose (Glc), oligomcyin (Oligo), and 2-DG. N = 4. (**B**,**E**) Glycolysis represents the ECAR after glucose addition less the non-glycolytic acidification, and the glycolytic capacity refers to the maximal ECAR in the presence of oligomycin less the non-glycolytic acidification. N = 4. (**C**,**F**) L-lactate secretion was determined by measuring its absolute concentration in the cell culture supernatant using a photometric assay. N = 7/3. All values are depicted as bar diagrams with the mean ± SEM and individual data points as open circles. Data points refer to monocytes sorted from buffy coats of individual donors. Statistical analysis was performed by one-way ANOVA followed by Tukey’s multiple comparison test. Levels of significance: *: *p* < 0.05; ***: *p* < 0.001; n.s.: *p* > 0.05.

Monocytes can fuel their energy demand from different sources. To embark on the analysis of this process in differentially activated human monocytes, we first investigated their immunometabolism using a Seahorse-based glycolysis stress test. Monocytes were either stimulated with LPS alone or together with 10^−5^ M Dex, and subsequently, the ECAR was monitored over time. Addition of glucose enhanced the pH change, which was further reinforced by oligomycin and reduced to baseline levels again by 2-DG (Figure 2A). The glycolytic function, resulting in a conversion of pyruvate to L-lactate, was higher in LPS-treated monocytes than in control monocytes, an effect which was partially reversed by Dex (Figure 2B). In support of this finding, we detected increased levels of L-lactate in the supernatant of LPS-treated monocytes, which were diminished by Dex as well (Figure 2C). Subsequently, we tested the glycolytic function of monocytes following stimulation with IL-4/13 (Figure 2D). In this case, the ECAR was unchanged, and Dex also did not have any effect (Figure 2E). This suggests that glycolysis and the glycolytic capacity in alternatively activated monocytes remain constant. In line with the Seahorse stress test, L-lactate levels were neither influenced by treatment with IL-4/13 nor in combination with Dex (Figure 2F). Collectively, the regulation of the glycolytic pathway in human monocytes by GCs is fundamentally different after stimulation with LPS or IL-4/13.

### 3.3. Transcriptional Control of the Glycolytic Pathway

**Figure 3:**
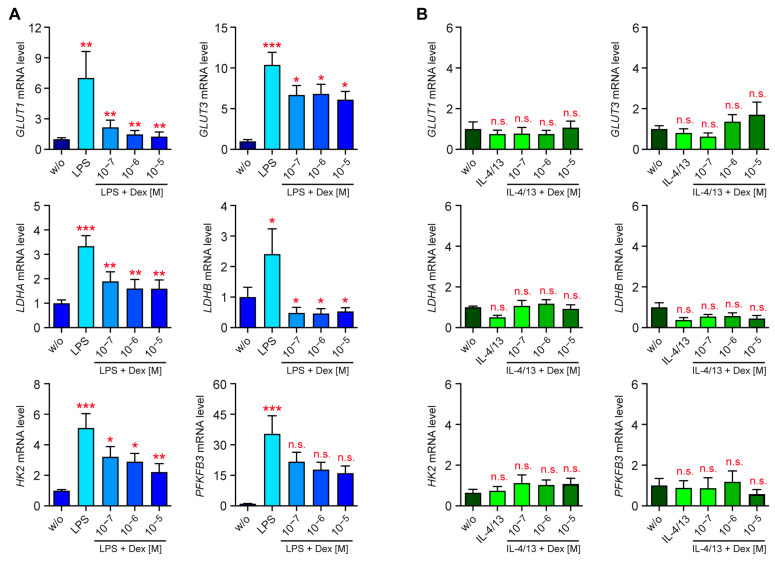
Regulation of metabolic gene expression in human monocytes. Monocytes were sorted from buffy coats of anonymous healthy individuals and activated in vitro for 20 h with (**A**) LPS or (**B**) IL-4 and IL-13, either in the absence or presence of 10^−7^ to 10^−5^ M Dex. Unstimulated monocytes (w/o) served as a control. Gene expression of *GLUT1*, *GLUT3*, *LDHA*, *LDHB*, *HK2*, and *PFKFB3* (N = 9–19) was analyzed by using RT-qPCR. *RNA18S* was used as a housekeeping gene; average mRNA levels in control monocytes were set to 1. All values are depicted as the mean ± SEM. Biological replicates refer to individual donors. Statistical analysis was performed using a one-way ANOVA followed by Tukey’s multiple comparison test. Levels of significance (from left to right: w/s vs. activated; activated vs. 10^−7^ M; activated vs. 10^−6^ M; activated vs. 10^−5^ M): *: *p* < 0.05; **: *p* < 0.01; ***: *p* < 0.001; n.s.: *p* > 0.05.

GCs can regulate the metabolic function of monocytes by controlling gene expression and non-genomic mechanisms. To address this issue, we performed an RT-qPCR analysis of monocytes that had been treated with LPS or IL-4/13 in the absence or presence of ascending concentrations of Dex. In agreement with the enhanced glycolytic function of classically activated monocytes, LPS caused an upregulation of the glucose transporter genes *GLUT1* and *GLUT3* as well as genes encoding the glycolytic enzymes *HK2*, *PFKFB3*, *LDHA*, and *LDHB* (Figure 3A). Dex counteracted these effects, albeit to a different degree and not always reaching significance (Figure 3A). Importantly, expression of the metabolic genes analyzed above remained unaltered after alternative activation by IL-4/13, and Dex also did not have any effect on their expression levels (Figure 3B). These findings suggest that regulation of the glycolytic pathway in human monocytes involves transcriptional mechanisms and confirm that it is only altered following classical but not alternative activation.

### 3.4. GCs Control Glycolysis in Monocytes via mTORC1

**Figure 4:**
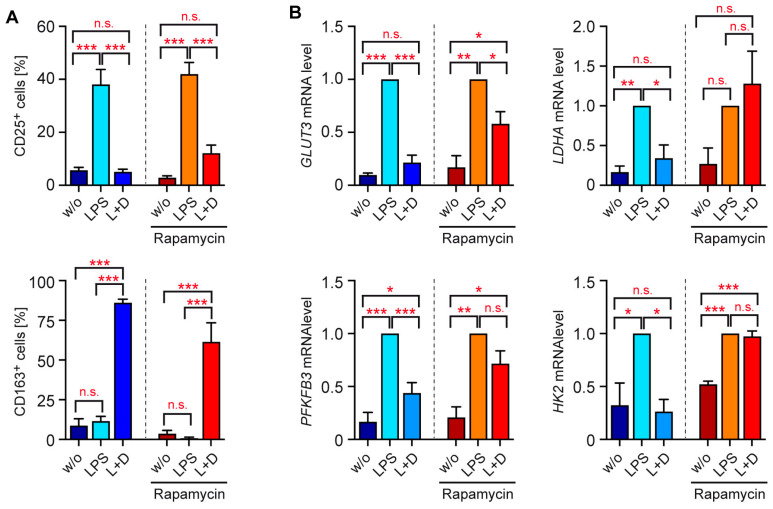
Impact of rapamycin on human monocytes. Monocytes were sorted from buffy coats of anonymous healthy individuals and activated in vitro for 20 h with LPS in the absence or presence of 10^−5^ M Dex. Unstimulated monocytes (w/o) served as a control. Addition of rapamycin was used to investigate the impact of mTORC1 signaling. (**A**) The percentage of HLA-DR^+^ CD14^+^ monocytes expressing CD25 or CD163 on the surface was determined by flow cytometry. N = 4. (**B**) Gene expression of *GLUT3*, *LDHA*, *PFKFB3*, and *HK2* was analyzed by RT-qPCR using *RNA18S* as a housekeeping gene; in each set of experiments, mRNA levels in LPS-treated monocytes were individually set to 1. N = 3–4. All values are depicted as the mean ± SEM; biological replicates refer to individual donors. Statistical analysis was performed using a one-way ANOVA followed by Tukey’s multiple comparison test. Levels of significance: *: *p* < 0.05; **: *p* < 0.01; ***: *p* < 0.001; n.s.: *p* > 0.05.

LPS is known to increase the glycolytic activity of monocytes via mTORC1, resulting in enhanced metabolic gene expression through the activation of transcription factors such as HIF1α, MYC, and SREBP [32]. To determine whether the repressive effect of Dex on glucose transporters and glycolytic enzymes in monocytes stimulated with LPS involves interference with this pathway, we employed the mTORC1 inhibitor rapamycin. Initially, flow cytometric analysis of classically activated monocytes was repeated but this time in the absence or presence of 20 nM rapamycin (Figure 1A and Figure 4A). Importantly, rapamycin neither influenced regulation of CD25 nor CD163 by Dex, confirming that the monocytes’ phenotype was independent of mTORC1 signaling (Figure 4A). Next, we investigated the control of glycolytic genes. *GLUT3*, *HK2*, *PFKFB3*, and *LDHA* were upregulated by LPS and suppressed by Dex as shown before (Figure 3A and Figure 4B). In the presence of rapamycin, however, Dex failed to repress expression of these genes (*LDHA*, *PFKFB3*, *HK2*) or it at least inhibited them less efficiently (*GLUT3*) (Figure 4B). This finding strongly suggests that interference of GCs with the glycolytic function in classically activated monocytes involves the mTORC1 pathway.

**Figure 5:**
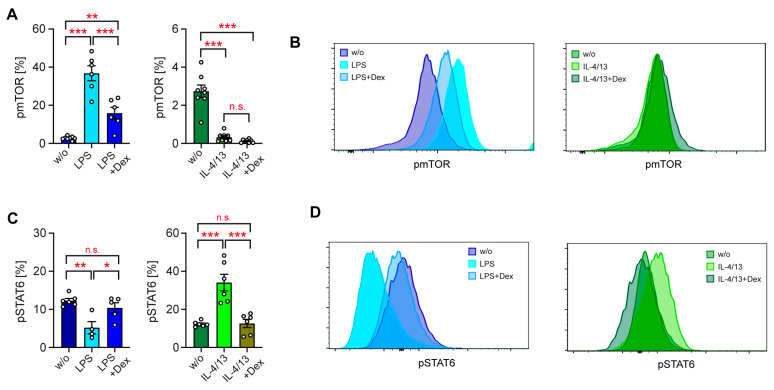
Regulation of intracellular signaling in human monocytes. Monocytes were sorted from buffy coats of anonymous healthy individuals and activated in vitro for 20 with LPS or IL-4 and IL-13, either in the absence or presence of 10^−5^ M Dex. Unstimulated monocytes (w/o) served as a control. Phosphorylation of signaling molecules was determined by intracellular flow cytometry. (**A**) The percentage of HLA-DR^+^ CD14^+^ monocytes containing pmTOR is depicted as bar diagrams, with the mean ± SEM and individual data points as open circles. N = 6–8. (**B**) Exemplary overlay histograms of pmTOR stainings of monocytes. (**C**) The percentage of HLA-DR^+^ CD14^+^ monocytes containing pSTAT6 is depicted as bar diagrams, with the mean ± SEM and individual data points as open circles. N = 4–6. (**D**) Exemplary overlay histograms of pSTAT6 stainings of monocytes. Data points refer to monocytes sorted from individual buffy coats. Statistical analysis was performed using a one-way ANOVA followed by Tukey’s multiple comparison test. Levels of significance: *: *p* < 0.05; **: *p* < 0.01; ***: *p* < 0.001; n.s.: *p* > 0.05.

GCs are known to interact with various cytosolic signaling pathways [1]. Hereby, GCs indirectly impact the activity of transcription factors such as HIF1α, MYC, and SREBP, which are induced by LPS via mTORC1 [32]. Similarly, GCs can also use this mechanism to regulate the activity of the transcription factor STAT6, which is phosphorylated by JAK proteins in response to IL-4 and IL-13. To address this issue, we initially investigated mTOR phosphorylation in monocytes. LPS enhanced pmTOR levels, which was largely prevented by Dex. In contrast, IL-4/13 reduced pmTOR, an effect which was unaltered by concomitant Dex treatment (Figure 5A,B). As a control, we also investigated regulation of STAT6 phosphorylation by GCs [33,34]. IL-4/13 increased pSTAT6 levels, LPS reduced them, and both effects were reversed by Dex (Figure 5C,D). In summary, our data suggest that GCs exert their suppressive activity on the glycolytic function of monocytes at least in part by inhibiting mTOR phosphorylation, thereby indirectly inhibiting metabolic gene expression. The observation that GCs repress pmTOR phosphorylation exclusively in classically activated monocytes presumably explains why metabolic gene expression in alternatively activated monocytes does not respond to GC treatment.

### 3.5. Mitochondrial Function of Activated Human Monocytes

**Figure 6:**
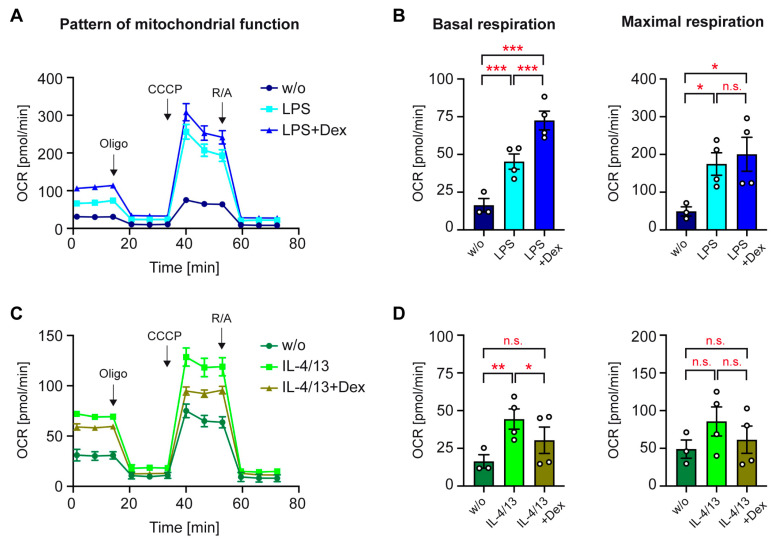
Regulation of mitochondrial function in human monocytes. Monocytes were sorted from buffy coats of anonymous healthy individuals and activated in vitro for 20 h with LPS or IL-4 and IL-13, either in the absence or presence of 10^−5^ M Dex. Unstimulated monocytes (w/o) served as a control. (**A**,**C**) The pattern of mitochondrial function was analyzed by measuring the OCR using a Seahorse XF96e Analyzer after sequential addition of oligomcyin (Oligo), CCCP, and rotenone/antimycin A (R/A). N = 3–4. (**B**,**D**) Basal respiration represents the OCR before addition of oligomycin minus the non-mitochondrial oxygen consumption, while maximal respiration refers to the OCR after adding CCCP minus the non-mitochondrial oxygen consumption. N = 3–4. All values are depicted as bar diagrams, with the mean ± SEM and individual data points as open circles. Data points refer to monocytes sorted from individual buffy coats. Statistical analysis was performed using a one-way ANOVA followed by Tukey’s multiple comparison test. Levels of significance: *: *p* < 0.05; **: *p* < 0.01; ***: *p* < 0.001; n.s.: *p* > 0.05.

OXPHOS is an efficient metabolic pathway to supply monocytes with energy and depends on the provision of pyruvate, mostly produced via the glycolytic pathway. To study the regulation of this process in activated monocytes, we analyzed the OCR using a Seahorse-based mitochondrial stress test. LPS served to achieve classical activation in the absence or presence of Dex. Mitochondrial respiration in the presence of glucose was first inhibited with oligomycin, and subsequently, maximal respiration was achieved by uncoupling the respiratory chain via adding CCCP (Figure 6A). Treatment with LPS increased basal and maximal respiration, and both effects were potentiated by the addition of Dex (Figure 6B). When we analyzed mitochondrial function of monocytes treated with IL-4/13, it turned out that this metabolic pathway was also induced after alternative activation. Basal respiration was significantly higher after treatment with IL-4/13 than in control monocytes and partially reduced by Dex (Figure 6C,D). Maximal respiration followed the same pattern of regulation although the differences were smaller and did not reach significance. The magnitude of basal respiration was comparable following stimulation with LPS or IL-4/13, whereas maximal respiration in classically activated monocytes was higher compared to alternatively activated ones (Figure 6B,D). We conclude that OXPHOS is reinforced by both stimuli, while the effects of GCs on this metabolic process fundamentally differ contingent upon monocyte polarization.

### 3.6. Dependency of Activated Human Monocytes on OXPHOS and Glucose

**Figure 7:**
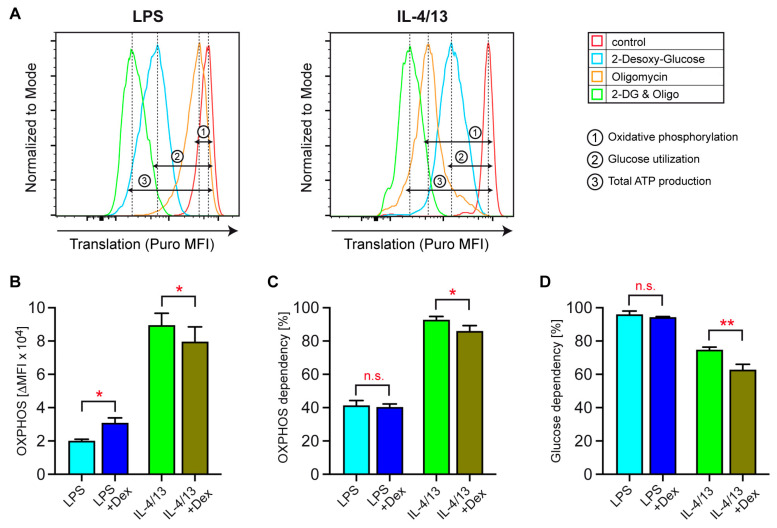
Dependency of human monocytes on OXPHOS and glucose. Monocytes were sorted from buffy coats of anonymous healthy individuals and activated for 20 h with LPS or IL-4 and IL-13, either in the absence or presence of 10^−5^ M Dex. Analysis by SCENITH assay was performed by incubating the cells with or without 2-DG and/or oligomycin for 15 min followed by the addition of puromycin for another 30 min. The cells were then extracellularly stained for HLA-DR followed by intracellular staining of peptidyl puromycin as a marker of translation. (**A**) Exemplary overlay histograms are depicted for HLA-DR^+^ monocytes activated by LPS or IL-4/13. ATP production by OXPHOS or glucose utilization as well as total ATP production are indicated in the plots. (**B**) Energy production by OXPHOS is depicted as the difference in the mean fluorescence intensities (MFIs) of peptidyl puromycin, as indicated in panel A. (**C**) Dependency on OXPHOS, calculated as the ratio of OXPHOS to total ATP production. (**D**) Dependency on glucose, calculated as the ratio of glucose utilization to total ATP production. N = 3 (individual buffy coats). All values are depicted as bar diagrams, with the mean ± SEM. Statistical analysis was performed by paired *t*-test. Levels of significance: *: *p* < 0.05; **: *p* < 0.01; n.s.: *p* > 0.05.

Seahorse-based stress tests are restricted to the analysis of bulk cell preparations and do not allow for drawing any conclusions concerning the relative dependency of single cells on individual metabolic pathways and substrates. Hence, we used a SCENITH assay to obtain further insights into energetic features of monocytes activated with LPS or IL-4/13 in the presence of absence of Dex. Labeling with puromycin allowed us to measure translational activity by intracellular flow cytometry as a surrogate marker of ATP production, whereas treatment with 2-DG and/or oligomycin enabled its assignment to individual metabolic processes (Figure 7A). Initially, the impact of GCs on the monocytes’ total energy production was determined on the basis of the signal intensity of peptidyl puromycin. In classically activated monocytes, ATP/GTP synthesis increased by 39 ± 10% (N = 3) after concomitant Dex treatment, whereas it remained almost constant in alternatively activated monocytes. This finding surprisingly suggests that GCs broadly reinforce metabolism after LPS stimulation, which is not the case in response to IL-4/13.

The amount of energy generated via OXPHOS was determined by quantification of peptidyl puromycin in the absence or presence of oligomycin (Figure 7A,B). OXPHOS was much less active in monocytes stimulated with LPS as compared to IL-4/13 (Figure 7B). In accordance with our Seahorse results, Dex augmented OXPHOS in classically activated monocytes, while it suppressed it in alternatively activated ones (Figure 7B). This confirms that GCs exert opposing effects on OXPHOS contingent upon monocyte polarization. Next, we evaluated the dependency of human monocytes on mitochondrial respiration. Classically activated monocytes generated only 40% of their ATP by OXPHOS, whereas alternatively activated monocytes covered more than 90% of their energy demand by this metabolic pathway (Figure 7C). Dependency of LPS-stimulated monocytes on OXPHOS was unaffected by GCs, while it was reduced in monocytes stimulated with IL-4/13 (Figure 7C). Moreover, we found that classically activated monocytes almost exclusively utilized glucose for ATP production, irrespective of Dex treatment (Figure 7D). In contrast, alternatively activated monocytes relied on glucose utilization to a lesser extent, which was further diminished by GCs (Figure 7D). Collectively, our data question the assumption that OXPHOS is a major target of metabolic regulation by GCs and rather support a model in which glycolysis is the primary rheostat of immunometabolism.

## 4. Discussion

**Figure 8:**
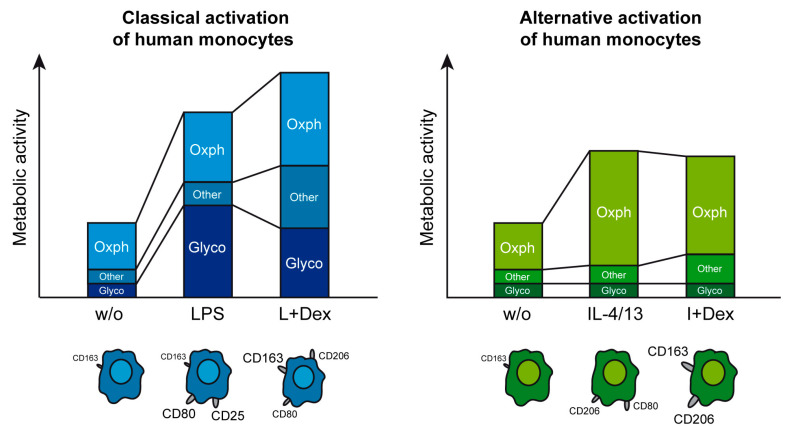
GC regulation of immunometabolism in classically and alternatively activated human monocytes. Scheme depicting the phenotype associated with each metabolic state and the assumed contribution of OXPHOS, lactate production via the glycolytic pathway, and ATP production independently of electron carriers (“other”) to the metabolic activity of human monocytes.

Monocytes play a crucial role in inflammation and homeostasis [4]. They can differentiate into macrophages in a matter of days, but at least in acute inflammation, they also fulfill essential functions by themselves. Contingent upon the presence of cytokines, microbial compounds, and hormones in the microenvironment, monocytes rapidly commit to different phenotypes endowing them with specific properties. Polarization comes along with a characteristic expression pattern of surface markers and is accompanied by metabolic adjustments that optimize the energy supply [15]. Our analysis of human monocytes confirmed discrete phenotypic changes in response to classical and alternative activation, but it also unveiled species differences compared to murine cells. It is noteworthy that this is true for the analysis of the energy metabolism as well. Previous studies mostly performed with murine cells provided insights into the energy metabolism of macrophages and the influence of GCs, but the reported data were partially contradictory and some open questions remained [25,26]. Here, we embarked to tackle them. Earlier work indicated that LPS increased glycolysis in macrophages, an effect which was inhibited by Dex. In contrast, conflicting results were reported with regard to mitochondrial function [25,26,27,28]. In most studies, LPS was found to decrease OXPHOS, whereas in others, an increase or no effect was observed. In some analyses, mitochondrial function was enhanced by concomitant treatment with Dex, while GCs failed to influence this metabolic pathway in others. We now demonstrate that LPS reinforces both glycolysis and OXPHOS in human monocytes. Furthermore, we show that GCs repress glycolysis while concomitantly enhancing OXPHOS, as revealed by Seahorse stress tests and a SCENITH assay (Figure 8). Most importantly, this finding indicates that GCs induce a metabolic switch in human monocytes and additionally promote ATP production in general rather than suppressing it. The observation that LPS fosters OXPHOS in human monocytes while it inhibits the same pathway in murine macrophages underscores that translation of results obtained in mouse models to humans is not always unrestrictedly possible.

To the best of our knowledge, we are the first to examine the immunometabolism of alternatively activated monocytes and its regulation by GCs. Our findings uncovered that IL-4/13 failed to influence glycolysis but promoted mitochondrial respiration to a comparable extent as LPS. Analysis by Seahorse stress tests and a SCENITH assay unveiled that Dex repressed OXPHOS but did not influence glycolysis. Hence, activation increases energy production of monocytes regardless of the stimulus, albeit by influencing metabolic pathways differently. LPS preferentially enhances the rapid glycolytic pathway, whereas IL-4/13 only reinforces OXPHOS, which represents the more sustained metabolic process. GCs induce a switch in both types of activated monocytes, albeit in opposite directions. In classically activated monocytes, the contribution of OXPHOS is increased by GCs, while the balance in alternatively activated monocytes is tipped in favor of glycolysis (Figure 8). It is noteworthy that the observed effects of Dex on metabolism are compatible with the suppression of monocyte function. Activation by LPS reflects a need for microbicidal molecules, inflammatory mediators, and antigen presentation. These processes all require the rapid provision of large amounts of energy equivalents, which is mainly achieved by upregulating glycolysis. Hence, inhibition of the glycolytic function is consistent with an anti-inflammatory activity of GCs, despite the enhanced OXPHOS. A microenvironment dominated by IL-4/13, however, is associated with a need for tissue repair and the termination of inflammation. In such a situation, an extensive energy supply is necessary too, although more persistently and less urgently, thus aligning with an engagement of the highly efficient mitochondrial pathway. Repression of OXPHOS in alternatively activated monocytes by GCs can thus be expected to even impede wound healing, as reported earlier [35]. In summary, GCs suppress monocyte function regardless of their polarization, albeit by influencing metabolic pathways differently.

Changes in metabolic gene expression observed in classically activated monocytes and the absence of transcriptional regulation in alternatively activated monocytes reflect their respective patterns of glycolytic activity. Although the majority of GC effects are thought to be mediated by GR binding to regulatory elements in target genes, an interference of the GR with cytosolic signaling pathways has been described as well [36,37]. As a matter of fact, LPS induced mTOR phosphorylation at serine 2448, which was inhibited by Dex, indicating that non-genomic mechanisms were involved in the regulation of energy metabolism of human monocytes. Since Dex failed to efficiently suppress gene expression of glucose transporters and glycolytic enzymes in the presence of rapamycin, it is fair to assume that GCs presumably inhibit glycolytic function by reducing mTOR phosphorylation similarly to activated T cells and thus influence metabolic gene expression indirectly [24]. In contrast, the regulation of OXPHOS in human monocytes remains intriguing. It has been previously shown for classically activated murine macrophages that GCs enhance the activity of pyruvate dehydrogenase via non-genomic mechanisms [25]. As a consequence, acetyl-CoA synthesized from pyruvate is fueled into the citric acid cycle and thereby promotes OXPHOS, an effect which is potentiated by inhibition of lactate dehydrogenase. However, in alternatively activated human monocytes, OXPHOS is repressed by GCs, indicating that different mechanisms must be at work dependent on polarization. A possible explanation for this discrepancy is provided by the previous observation that GCs inhibit OXPHOS in muscle and liver as well. Here, pyruvate dehydrogenase kinase 4 is induced by GCs, thereby diminishing the conversion of pyruvate to acetyl-CoA. As a consequence, mitochondrial respiration is inhibited, resulting in increased fatty acid oxidation and gluconeogenesis [38]. Taken together, the available data suggest that regulation of OXPHOS by GCs is highly cell-type-specific.

It is indisputable that GCs influence the immunometabolism of myeloid cells. However, our study now surprisingly uncovered that the dependency of classically activated monocytes on mitochondrial respiration was unaffected by GCs. Despite its higher activity, the relative contribution of OXPHOS to the cells’ provision with ATP was unaltered. This can only be explained by a parallel increase in ATP/GTP synthesis independent of electron carriers like NADH, thus compensating for the diminished glycolytic activity, resulting in lactate production (Figure 8). Processes that qualify for this condition are ATP synthesis during conversion of glucose or glycerol to acetyl-CoA and GTP production by succinyl-CoA synthase. In alternatively activated monocytes, OXPHOS dependency was reduced in response to GCs, which concurs with the general inhibition of this pathway. Nonetheless, one must still assume a minor increase in NADH-independent ATP/GTP synthesis after GC treatment of alternatively activated monocytes (Figure 8). Interestingly, the polarized monocytes also differed in their dependency on glucose, with classically activated ones almost exclusively relying on this substrate regardless of Dex treatment. In contrast, the reduced glucose dependency of alternatively activated monocytes was further promoted by Dex, which indicates reinforced energy production from fatty acids and amino acids.

## 5. Conclusions

In the present study, we uncovered fundamental differences in the control of energy metabolism by GCs in human monocytes contingent upon the activating stimuli. Classical and alternative activation reinforced the energy supply of human monocytes, albeit by differently regulating metabolic pathways (Figure 8). LPS stimulated both glycolysis and OXPHOS, whereas IL-4/13 stimulated only the latter. GCs induced a metabolic switch under each activating condition, albeit in opposite directions and at different magnitudes. In classically activated monocytes, repression of glycolysis was the dominant effect, while alternatively activated monocytes rather experienced an opposite switch characterized by the repression of mitochondrial respiration. These metabolic effects are mediated through a complex network of transcription factors acting in conjunction with upstream signaling pathways. Overall, our study supports the concept that the control of immunometabolism contributes to the anti-inflammatory activity of GCs, although this effect is not achieved by impeding the energy supply of myeloid cells in general but rather by readjusting the balance between individually metabolic pathways, in particular by targeting the glycolytic function.

## Data Availability

Data and material are available on reasonable request.

## Supplementary Materials

The following supporting information can be downloaded at: https://www.mdpi.com/article/10.3390/biom15101422/s1. Supplementary Table S1. Primer sequences.

## Abbreviations

The following abbreviations are used in this manuscript:

2-DG	2-desoxy-D-glucose
ECAR	extracellular acidification rate
GC	glucocorticoid
GR	glucocorticoid receptor
Dex	dexamethasone
OCR	oxygen consumption rate
OXPHOS	oxidative phosphorylation
SCENITH	single-cell energetic metabolism by profiling translation inhibition
