# Supplementary material for: Glucocorticoids Induce an Opposite Metabolic Switch in Human Monocytes Contingent upon Their Polarization
Source: Biomolecules. 2025 Oct 7;15(10):1422. doi: 10.3390/biom15101422 (PMC12564316; doi:10.3390/biom15101422)
Supplement: Supplementary file 1 [file biomolecules-15-01422-s001.zip › biomolecules-3887028-supplementary.pdf]

Supplementary Table S1. Primer sequences

Gene	Forward primer	Reverse primer
RNA18S	5'-TCC AGG TCT TCA CGG AGC TTG TT-3'	5'-GGA TGT AAA GGA TGG AAA ATA CA-3'
CD25	5‘-GTG GTG GGG CAG ATG GTT TA-3‘	5‘-TTG TGA CGA GGC AGG AAG TC-3‘
CD163	5‘-GCA GTT TCC TCA AGA GGA GAG AA-3‘	5‘-ATG GCC TCC TTT TCC ATT CCA-3‘
GLUT1	5′-TGG CAT CAA CGC TGT CTT CT-3′	5′-AGC CAA TGG TGG CAT ACA CA-3′
GLUT3	5′-GGA CGT GGA GAA AAC TTG CTG-3′	5′-TCA GAG CTG GGG TGA CCT TC-3′
HK2	5′-CCT CCC CTC TCG CGT CT-3′	5′-AGA GAT ACT GGT CAA CCT TCT GC-3′
LDHA	5′-ACG TGC ATT CCC GAT TCC TT-3′	5′-AAC AGC ACC AAC CCC AAC AA-3′
LDHB	5′-GCC TTC TCT CTC CTG TGC AA-3′	5′-CCT CTT CTT CCG CAA CTG GT-3′
PFKFB3	5′-CAG CTG CCT GGA CAA AAC AT-3′	5′-CGT CTG CCT CAG TGT TTC CT-3′
